# Unveiling microbial dynamics in lung adenocarcinoma and adjacent nontumor tissues: insights from nicotine exposure and diverse clinical stages via nanopore sequencing technology

**DOI:** 10.3389/fcimb.2024.1397989

**Published:** 2024-08-27

**Authors:** Kangli Yang, Shuaifeng Wang, Zheng Ding, Kai Zhang, Weiwei Zhu, Huifen Wang, Mengshu Pan, Xiangnan Li, Hongmin Wang, Zujiang Yu

**Affiliations:** ^1^ Department of Respiratory, The First Affiliated Hospital of Zhengzhou University, Zhengzhou, China; ^2^ Gene Hospital of Henan Province, Precision Medicine Center, The First Affiliated Hospital of Zhengzhou University, Zhengzhou, China; ^3^ Department of Thoracic Surgery, The First Affiliated Hospital of Zhengzhou University, Zhengzhou, China; ^4^ Department of Infectious Diseases, The First Affiliated Hospital of Zhengzhou University, Zhengzhou, China; ^5^ Department of Grassroots Medical, The Second Affiliated Hospital of Anhui Medical University, Hefei, China

**Keywords:** lung cancer, intratumor microbiota, metabolic pathways, nanopore sequencing, clinical stages, nicotine exposure

## Abstract

**Background:**

Lung is the largest mucosal area of the human body and directly connected to the external environment, facing microbial exposure and environmental stimuli. Therefore, studying the internal microorganisms of the lung is crucial for a deeper understanding of the relationship between microorganisms and the occurrence and progression of lung cancer.

**Methods:**

Tumor and adjacent nontumor tissues were collected from 38 lung adenocarcinoma patients and used nanopore sequencing technology to sequence the 16s full-length sequence of bacteria, and combining bioinformatics methods to identify and quantitatively analyze microorganisms in tissues, as well as to enrich the metabolic pathways of microorganisms.

**Results:**

the microbial composition in lung adenocarcinoma tissues is highly similar to that in adjacent tissues, but the alpha diversity is significantly lower than that in adjacent tissues. The difference analysis results show that the bacterial communities of *Streptococcaceae*, *Lactobacillaceae*, and *Neisseriales* were significantly enriched in cancer tissues. The results of metabolic pathway analysis indicate that pathways related to cellular communication, transcription, and protein synthesis were significantly enriched in cancer tissue. In addition, clinical staging analysis of nicotine exposure and lung cancer found that *Haemophilus*, *paralinfluenzae*, *Streptococcus gordonii* were significantly enriched in the nicotine exposure group, while the microbiota of *Cardiobactereae* and *Cardiobacterales* were significantly enriched in stage II tumors. The microbiota significantly enriched in IA-II stages were *Neisseriaeae*, *Enterobacteriales*, and *Cardiobacterales*, respectively.

**Conclusion:**

Nanopore sequencing technology was performed on the full length 16s sequence, which preliminarily depicted the microbial changes and enrichment of microbial metabolic pathways in tumor and adjacent nontumor tissues. The relationship between nicotine exposure, tumor progression, and microorganisms was explored, providing a theoretical basis for the treatment of lung cancer through microbial targets.

## 1 Introduction

Lung cancer, one of the most prevalent cancers globally, presents profound health challenges and societal burdens, surpassing the combined mortality rates of other major tumor types. Despite advancements in diagnostic techniques, coupled with resistance to conventional chemotherapy, the prognosis for lung cancer remains notably grim (Herbst et al., 2018). Its pathogenesis is intricately multifaceted, encompassing genetic mutations, epigenetic alterations, environmental influences, and disruptions in immune function. While comprehensive cancer genome maps have been unveiled for lung adenocarcinoma and lung squamous cell carcinoma, the extrinsic factors influencing lung cancer development remain elusive (Network, 2014; Campbell et al., 2016).

The human microbiota, regarded as an indispensable component of the body, has evolved into a distinct organ system within the human physiology. These diverse microbial populations inhabit various regions including the mouth, intestines, digestive tract, and skin (Matson et al., 2018; Tanoue et al., 2019), playing pivotal roles in maintaining human health (Clemente et al., 2012). Numerous studies have demonstrated discernible differences in the microbiome composition between individuals with good health and those afflicted by diseases, underscoring the profound impact of microorganisms on immune responses. While cancer is traditionally attributed to genetic and environmental factors, approximately 20% of human malignancies are linked to microorganisms, influencing tumor development (de Martel et al., 2012). Notably, the integration of pathogens such as EB virus, hepatitis B virus, hepatitis C virus, and human papillomavirus into the human genome heightens susceptibility to specific cancers, such as cervical cancer. Additionally, Helicobacter pylori bacteria colonization of the gastric mucosa is implicated in the onset of gastric cancer (Warren and Marshall, 1983; Marshall et al., 1985). Garrett et al. delineate three primary mechanisms through which microbiota contribute to tumor initiation and progression: (1) modulation of cell proliferation and apoptosis pathways, (2) modulation of the immune system and immune responses, and (3) modulation of host secretion factors, as well as metabolism of food and drugs (Garrett, 2015).

As with an organ for gas exchange, the lungs are directly exposed to the external environment, allowing for the exchange of microorganisms between the lungs and the oral cavity. Researchers once believed that healthy lungs were sterile, as traditional microbial culture methods failed to isolate and cultivate bacteria from the lower respiratory tract. However, advancements in high-throughput sequencing technology have revealed that the lungs harbor a diverse bacterial community, regardless of health status (Dickson et al., 2016). This lung microbiota is characterized by its complexity, comprising a variety of bacterial species. The main phyla found in the lung microbiome include Firmicutes, Proteobacteria, Bacteroidetes, and Actinobacteria (Moffatt and Cookson, 2017). At the genus level, *Prevotella*, *Vibrio*, *Streptococcus*, *Neisseria*, *Haemophilus*, *Clostridium*, *Sphingomonas*, *Pseudomonas*, *Acinetobacter*, and *Megacoccus* are among the predominant genera, while *Staphylococcus* and *Corynebacterium* dominate the airway microbiota (Hilty et al., 2010; Erb-Downward et al., 2011; Gomes et al., 2019).

The use of 16S sequencing technology has led to a growing body of evidence linking local ecological imbalances to cancer. For instance, studies comparing lung tumor tissue samples with non-malignant lung tissue have shown significantly lower alpha diversity in the microbial community of lung tumors, indicating a correlation with cancer staging (Yu et al., 2016). Furthermore, it has been observed that *Thermus* is more abundant in tumor tissue from advanced patients, whereas *Legionella* is more prevalent in patients with metastasis, suggesting these bacteria may play a role in lung cancer progression (Yan et al., 2015). Some studies propose that microbial communities in saliva, bronchoalveolar lavage fluid, and sputum samples could potentially serve as biomarkers for predicting cancer occurrence (Yan et al., 2015; Lee et al., 2016; Cameron et al., 2017).

In studies on lung adenocarcinoma, researchers have discovered that indigenous microorganisms can penetrate the resident lung cells, triggering inflammation associated with the disease via γδ T cells. This inflammatory response is potentially initiated by symbiotic bacteria, prompting bone marrow cells to produce Myd88-dependent IL-1β and IL-23. Consequently, this cascade induces the proliferation and activation of Vγ6+Vδ1+ γδ T cells, leading to the secretion of IL-17 and other effector molecules, thus promoting inflammation and the proliferation of tumor cells (Jin et al., 2019).

This study involved the collection of paired tumors and adjacent nontumor tissues samples from 33 patients undergoing lung adenocarcinoma surgery. Employing high throughput 16S sequencing technology, we analyzed the Intratumoral microbiota. Our findings revealed that the genera *Haemophilus*, *Rhodopseudomonas*, *Salmonella*, and *Pseudomonas* constituted the predominant microbial communities in the lungs, comprising over 99% of the total microbial population. Furthermore, the cancer tissues exhibited higher overall microbial diversity compared to adjacent tissues, with notable enrichment of *Streptococcaceae*, *Lactobacillaceae*, and *Neisseriales* in cancerous tissues. Additionally, we examined the correlation between nicotine exposure, tumor clinical staging and intratumoral microbiota. The microorganisms identified in tumor tissues in our study offer a novel avenue for the clinical treatment of lung adenocarcinoma through microbial interventions.

## 2 Materials and methods

### 2.1 Sample collection

In this study, we collected data from 38 patients with primary lung cancer diagnosed for the first time at the First Affiliated Hospital of Zhengzhou University between October 2022 and June 2023. After excluding five unpaired samples, we analyzed a final set of 33 paired samples. All subjects signed informed consent forms before surgery, and this project was approved by the Ethics Committee of the First Affiliated Hospital of Zhengzhou University (2022-KY-0677-003). The inclusion criteria were as follows: newly diagnosed stage II lung adenocarcinoma patients who had not undergone radiotherapy or chemotherapy, agreed to participate in the research, and had no history of other organ tumors or intestinal-related surgery. Exclusion criteria included the presence of bronchiectasis or interstitial lung disease revealed by chest CT examination, COPD indicated by pulmonary function testing, a history of asthma, use of antibiotics or steroids for treatment within the past 3 months, and concurrent pneumonia diagnosed through serological and imaging examinations. For detailed clinical information, please refer to **Supplementary Table 1**.

### 2.2 DNA extraction, library construction, and high-throughput sequencing

The Zymo Research BIOMICS DNA Microprep Kit (Cat # D4301) was used for microbial gDNA purification. The integrity of gDNA was detected by 0.8% agarose gel electrophoresis, followed by nucleic acid concentration detection using Tecan F200 (PicoGreen dye method). Use specific primers with a Barcode full-length of 16S to amplify the designated region of the sample, The primer information is as follows: (8F: 5’AGAGTTTGATCATGGCTCAG3’; 1492R: 5’CGGTTACCTTGTTACGACTT3’). Each sample undergoes 3 replicates, and each PCR reaction terminates at the linear amplification stage. After PCR, the PCR products of the same sample were mixed and subjected to electrophoresis detection. The PCR products were cut and recovered using a gel recovery kit, and the target DNA fragments were washed and recovered using TE buffer. The PCR recovered products were detected and quantified using Qubit 2.0 (Thermo Fisher, Inc., USA), and after passing the quality control, the Nanopore R9.4.1 library kit was used for machine library construction. The library was sequenced using MinION sequencer and real-time high-precision base calling was performed.

### 2.3 Data quality control and sequence annotation analysis

The raw data obtained from sequencer were filtered by qcat and NanoFILT tools to obtain high-quality target sequences for subsequent analysis (De Coster et al., 2018). To obtain the classification information corresponding to each sequence, the classification based on *k-mer* matching was used to annotate the classification of all sequences. Usually includes three steps: first, cut the sequence into several k-mers; second, compare the *k-mer* to the species classification database to obtain its LCA (Least Common Ancestor) and the number of comparisons made; third, construct a component class tree based on the above data, and then calculate the sum of weights for each root to leaf route on the classification tree, with the maximum being the classification level of the sequence. Finally, merge the same species (OTUs) for statistical analysis. Functional prediction is a linear prediction based on the microbial functional gene profile in the KEGG database(https://www.kegg.jp).

### 2.4 Alpha diversity and beta diversity analysis methods

Alpha diversity is analyzed using R language for statistical analysis, the PD index is calculated using the Picante package, and other indices are calculated using the Vegan package. Use GuniFrac package to calculate Unifrac distance, and use Vegan package’s vegdits function to calculate Bray Curtis and Jaccard distance. PCoA analysis uses the Ape package. PCA and NMDS analysis were conducted using the vegan package. Cluster analysis uses the hclust function of the STAS package. The calculations of Anosim and PerMANOVA use the anosim and adonis functions of the vegan package.

### 2.5 Differential analysis and biomarker analysis

LEfSe, an abbreviation for Linear discriminant analysis Effect Size, is a robust data analysis method employed to assess the impact of species abundance on differential effects. This algorithm places significant emphasis on both statistical significance and biological relevance. Implementation of LEfSe is facilitated through the Microbiomarker R package, and the web-based platform (http://huttenhower.sph.harvard.edu) is also widely utilized for LEfSe analysis. In this study, a threshold of LDA>2 was considered indicative of both statistical and biological significance. For statistical and visualization analyses of community functional differences, STAMP software was utilized. Additionally, the Wilcoxon rank-sum test was employed to analyze inter-group differences in this study.

### 2.6 Data visualization and statistical analysis

The images and statistical analysis results presented in the article were generated using R software (version 4.2.2). The Kruskal-Wallis rank sum test for two groups was conducted using the Kruskal. Test function. Furthermore, the one-way Welch t-test method was applied to compare differences between the two groups. Multiple group disparities were examined through one-way analysis of variance (ANOVA) and LSD. Permanent multivariate analysis of variance (PERMANOVA) and redundancy analysis (RDA) were employed to evaluate the impact of patient phenotype on microbiota composition. Distinct lowercase letters were utilized to denote significant differences. Spearman rank correlation test was used to explore relationships between microbiota composition and clinical factors, with a heatmap generated using the pheatmap package. Asterisks (*) denote statistical significance, with * representing *p* < 0.05 and ** representing *p* < 0.01. Additional visualizations were created using ggplot2, the EasyMicroPlot software package, or the MicrobiotaProcess software package.

## 3 Results

### 3.1 Clinical information statistical analysis of lung cancer patients

We examined the baseline clinical characteristics of lung adenocarcinoma patients and observed a notable disparity: there were significantly fewer individuals with upper lung tumors compared to those with lower lung tumors within the smoking population. While the incidence of cancer was higher among males in the smoking cohort than females, this discrepancy can be attributed to the relatively low prevalence of smoking among Chinese women, rather than indicating any inherent biological significance (see **Table 1**). Moreover, we found no statistically significant differences in other clinical indicators across the groups.

**Table 1:** Demographic and clinical characteristics of the cohort.

Characteristic	Smoke			Stage			
	no, N = 30*^1^*	yes, N = 8*^1^*	*P^2^*	IA, N = 22*^1^*	IB, N = 11*^1^*	II, N = 5*^1^*	*P^3^*
age			0.616				0.751
Mean (SD)	M S 59.43 (8.50)	60.25 (5.09)		58.73 (7.46)	60.82 (9.14)	60.80 (7.69)	
Median (IQR)	MI 57.50 (53.25, 65.75)	60.00 (56.50, 62.75)		59.00 (54.00, 63.50)	57.00 (55.00, 68.50)	58.00 (57.00, 63.00)	
Range	R 45.00, 74.00	54.00, 69.00		45.00, 74.00	45.00, 73.00	53.00, 73.00	
gender	F 25 (83%) M 5 (17%)	F 0 (0%) M 8 (100%)	<0.001	F 12 (55%) M 10 (45%)	F 9 (82%) M 2 (18%)	F 4 (80%) M 1 (20%)	0.341
stage	IA 17 (57%) IB 9 (30%) II 4 (13%)	5 (63%) 2 (25%) 1 (13%)	>0.999				
smoke				5 (23%)	2 (18%)	1 (20%)	>0.999
location	lower 14 (47%) upper 16 (53%)	0 (0%) 8 (100%)	0.017	7 (32%) 15 (68%)	4 (36%) 7 (64%)	3 (60%) 2 (40%)	0.563
antibiotic			0.441				0.221
Aminoglycosides	3 (10%)	2 (25%)		3 (14%)	1 (9.1%)	1 (20%)	
Cephalosporins	26 (87%)	6 (75%)		19 (86%)	10 (91%)	3 (60%)	
Penicillin	1 (3.3%)	0 (0%)		0 (0%)	0 (0%)	1 (20%)	

^1^n (%).

^2^Wilcoxon rank sum test; Fisher’s exact test; Wilcoxon rank sum exact test.

^3^Kruskal-Wallis rank sum test; Fisher’s exact test.

### 3.2 Characteristics of microbial composition in tumor and adjacent nontumor tissues

The libraries were sequenced utilizing the nanopore MINION sequencer, and subsequent data from the machine were analyzed through the Silva database (https://www.arb-silva.de), a total of 1190 Operational Taxonomic Units (OUT) were identified, spanning 19 phyla, 40 classes, 99 orders, 212 families, 477 genera, and 933 species levels. For a more detailed classification, please refer to **Supplementary Table 2**.

**Figure 1:**
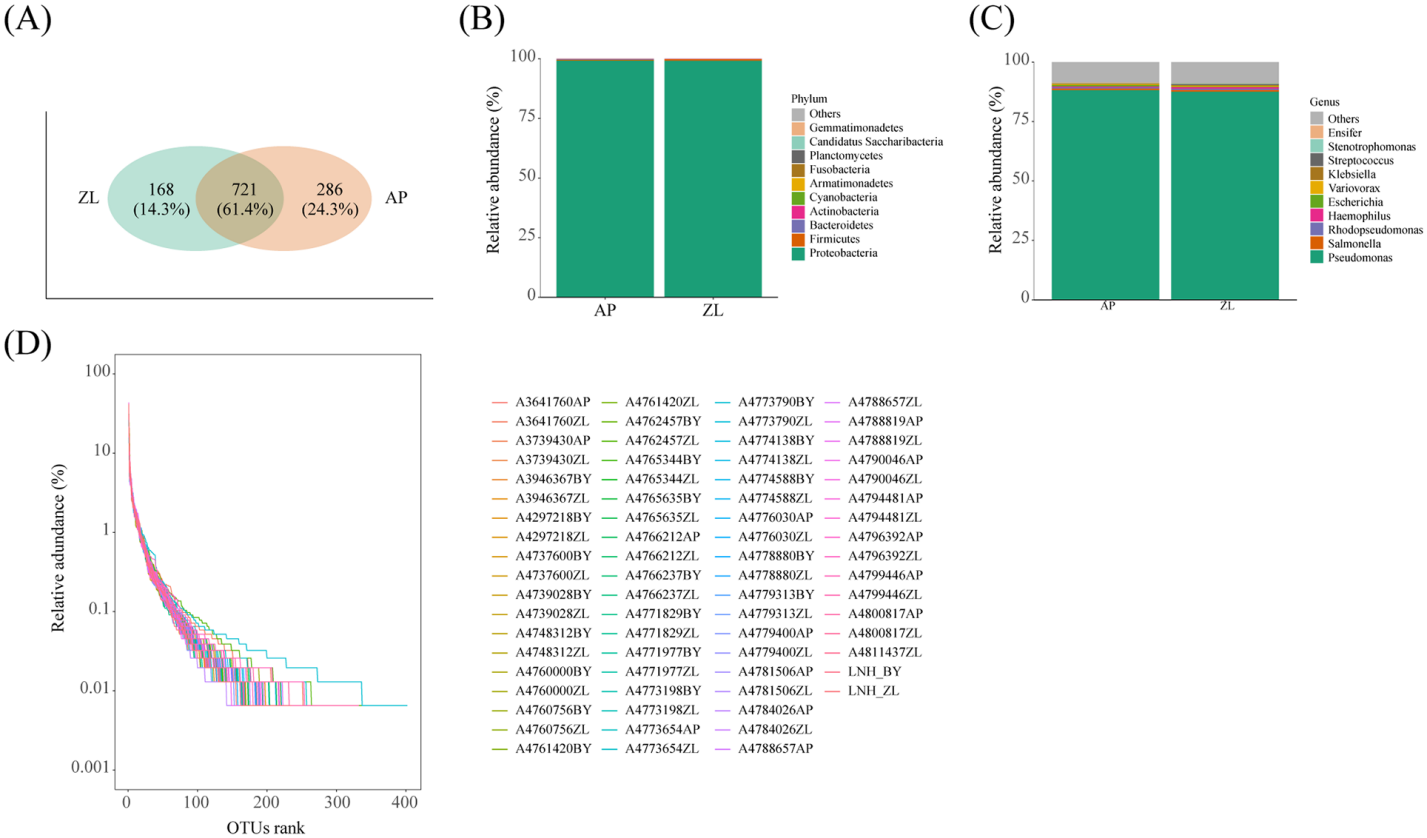
Microbial composition in tumor and adjacent nontumor tissues. **(A)** The Venn diagram shows the detection of common and unique OUT in cancer and adjacent nontumor tissues. **(D)** Rank Abundance curves of species in the sample. **(B, C)** Represents the proportion of microorganisms at the phylum and genus levels.

Initially, 1000 sequences were randomly selected from each sample for dilution curve analysis and Rank Abundance curve analysis. The results indicated that each sample underwent sequencing of more than 10,000 sequences, which is deemed adequate for microorganism identification within the sample. Notably, the Rank Abundance curve exhibited a steep decline, signifying a skewed distribution of microbial abundance and species evenness within lung tissue (**Figure 1D**).

Venn analysis revealed that 721 OUT were common to both tumor and adjacent nontumor tissues, constituting 61.4% of the total. Furthermore, there were 168 and 286 unique OUT identified in tumor and adjacent nontumor tissues, respectively (**Figure 1A**). Subsequent paired analysis of tumor and paracancerous species composition unveiled that the top four microorganisms with the highest proportion at the phylum level were *Cyanobacteria*, *Actinobacteria*, *Bacteroidetes*, and *Firmicutes*, alongside Proteobacteria. Similarly, at the genus level, the top four microorganisms with the highest proportion were *Haemophilus*, *Rhodopseudomonas*, *Salmonella*, and *Pseudomonas* (**Figures 1B, C**).

### 3.3 Differences in alpha and beta diversity between tumor and adjacent nontumor tissues

**Figure 2:**
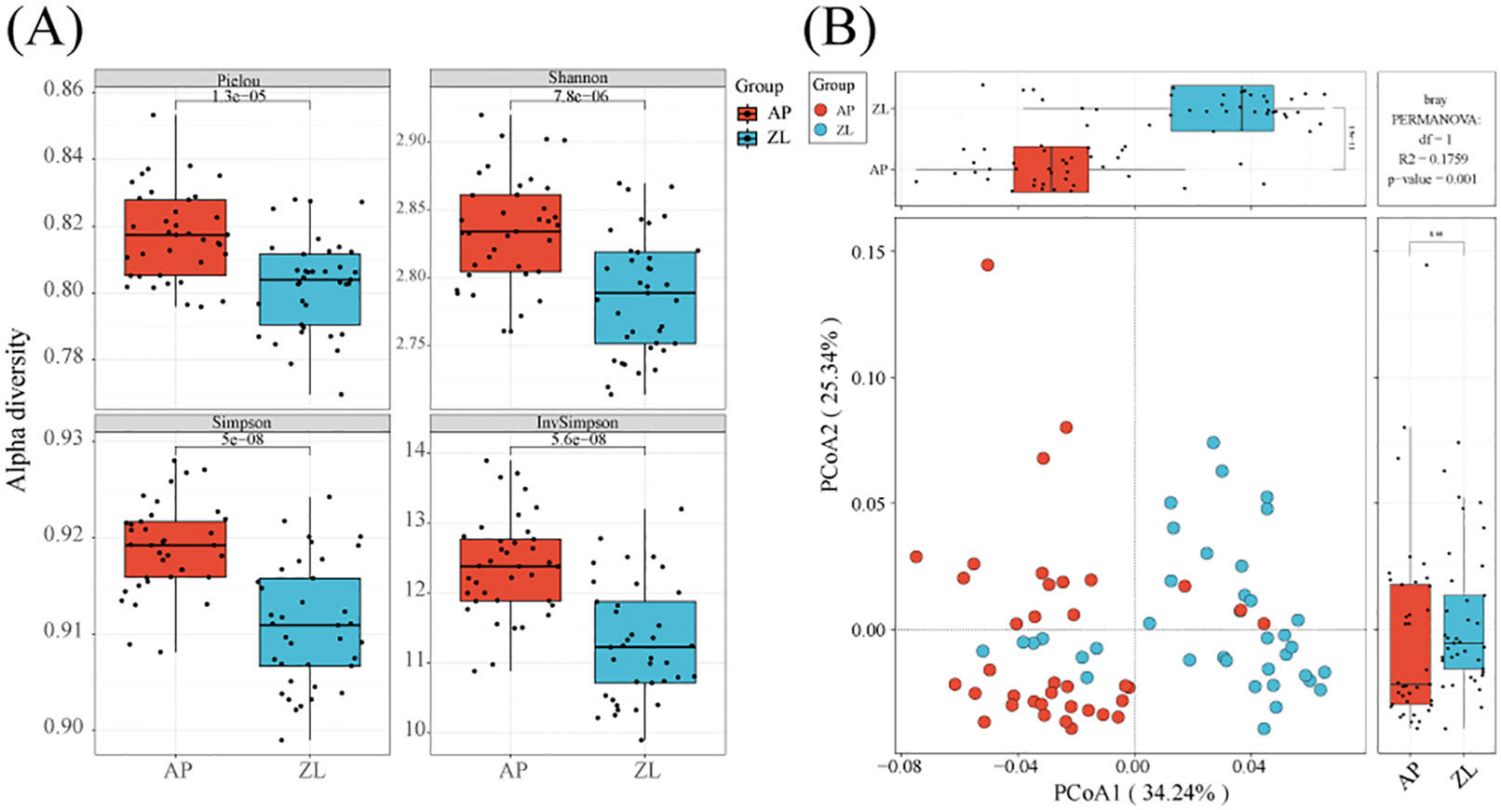
Alpha and beta diversity in tumor and adjacent tissues. **(A)** Box plots of differences in alpha diversity index between tumors and adjacent nontumor tissues. **(B)** PCoa analysis of microorganisms in tumors and adjacent nontumor tissues.

By calculating the Alpha diversity index, we can gain insights into the richness, evenness, and overall diversity of the microbial community in the samples. We initiated data analysis by applying specific filtering parameters: min-relative=0.001, min-ratio=0.7. Subsequently, we utilized Vega software to compute the Alpha diversity indices. The results revealed that the four indices of Alpha diversity (Pielou, Shannon, Simpson, Inv Simpson) were higher in adjacent nontumor tissues to those found in tumor tissues (**Figure 2A**). To ensure the robustness of our findings, we recalculated the Alpha diversity using adjusted filtering parameters: min-relative=0.001, min-ratio=0.5. Remarkably, the trends of the four indices remained consistent (**Supplementary Figure 1**).

Moreover, we employed PCoA (Principal Coordinates Analysis) with the Bray-Curtis dissimilarity metric to assess the beta diversity between tumor and adjacent nontumor groups. Our analysis demonstrated significant differences between the two groups, particularly evident on the PCoA1 coordinate axis (*P* = 1.9e-11). Furthermore, we conducted inter-group species differences analysis using PermANOVA (permanent multivariate analysis of variance), yielding a *p*-value=0.001, indicating significant dissimilarities between the tumor and adjacent cancer groups (**Figure 2B**).

### 3.4 Differences in microbial diversity between tumor and adjacent nontumor tissues

**Figure 3:**
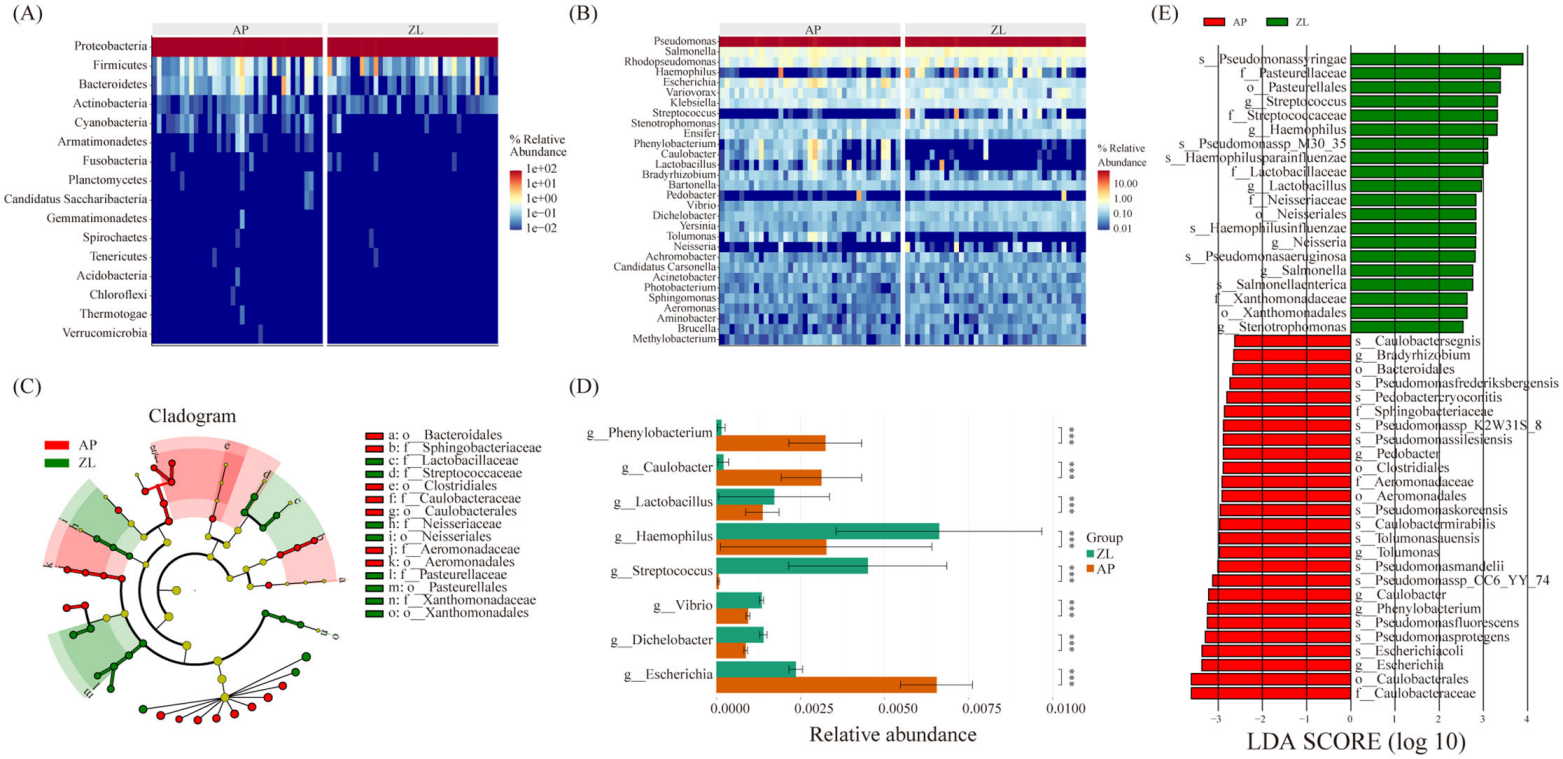
Microbial differences between cancer and adjacent tissues. **(A, B)** Heatmap of relative abundance of the top 20 microorganisms at the phylum and genus levels. **(C)** Caldogram of differential bacterial taxa from the phylum to the genus level. **(D)** Differences in microbes between groups at the genus level based on the wilcoxon rank sum test. **(E)** LDA histogram of differential microbiota at the genus level.

The microbial composition similarity between tumor and adjacent nontumor tissues was notably high, as depicted in the heatmap revealing substantial differences at the phylum level: *Cyanobacteria*, *Bacteroidetes*, *Armanimonades*, *Fusobacteria*, *Planctomycotes*, *Candidatus*, *Saccharibacteria* (**Figure 3A**). Additionally, at the genus classification level, there were marked distinctions among *Streptococcus*, *Haemophilus*, *Phenobacterum*, *Caulobacter*, *Lactobacillus*, and *Bradyrhizobium* (**Figure 3B**). Notably, the tumor group exhibits higher abundance of *Haemophilus* and *Streptococcus* compared to the adjacent group.

To discern significantly enriched bacteria in cancer and adjacent groups, we employed the LEfSe method, setting thresholds at LDA scores ≥ 2.5 and *P*-value < 0.05. The analysis revealed significant enrichment of *Lactobacillaceae*, *Streptococcaceae*, *Neisseriaceae*, and *Pasteurella* in tumor tissue (**Figures 3C, E**). Particularly, *Streptococcus*, *Haemophilus parainfluenzae*, *Lactobacillaceae*, and *Neisseria* displayed significantly increased abundance in the tumor group, demonstrating an association with tumors. Furthermore, non-parametric tests (rank sum tests) were applied to assess relative differences in bacteria between tumors and adjacent tissues. The abundance of Lactobacillus, Streptococcus, and Haemophilus in tumor tissues was found to be significantly higher than that in adjacent tissues, aligning with the findings from the LEFse analysis (**Figure 3D**; **Supplementary Table 3**).

### 3.5 Differences of function between Intratumoral microbes and adjacent tissue microbiome

The intricate interplay between microorganisms and hosts hinges on the release of metabolites, which interface with host cells and the immune microenvironment, precipitating shifts in host cell metabolism and immune modulation. These alterations can disrupt regular cellular processes such as proliferation, apoptosis, and potentially culminate in carcinogenesis.

**Figure 4:**
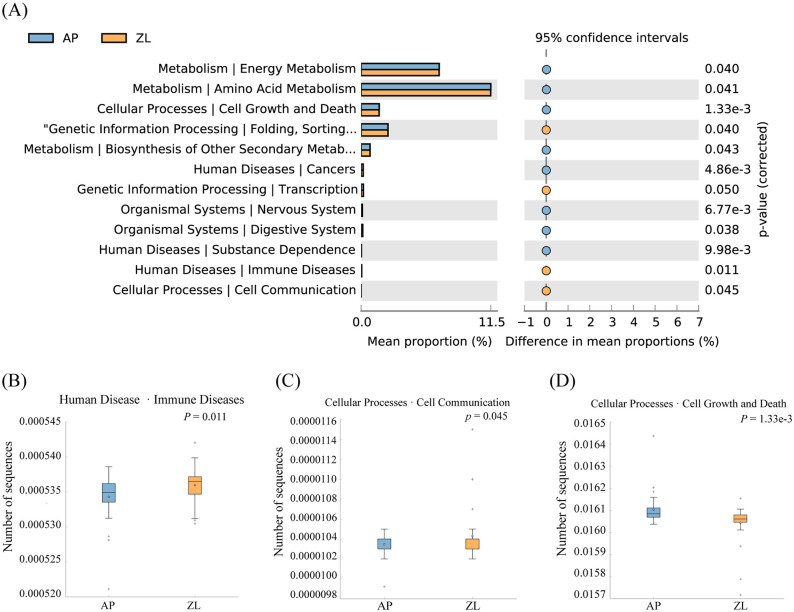
Differences in microbial metabolic pathways between cancer and adjacent groups. **(A)** Significantly enriched metabolic pathways by *p*-value ≤ 0.05. **(B)** Boxplot of Amino Acid Metabolism pathway in two groups. **(C)** Boxplot of Cell Growth and Death in two groups. **(D)** Boxplot of Folding, Sorting and Degradation pathway in two groups.

In our study, we utilized PICRUSt2 software to forecast the microbial KEGG pathways from the detected microbiome, complemented by statistical analyses of microbial metabolic pathways discrepancies between tumor and adjacent nontumor tissues groups conducted through STAMP software. Our findings revealed significant enrichments at the L2 level, with Folding, Sorting and Degradation, Transcription, Immune Diseases, and Cell Communication notably heightened in the tumor group (**Figure 4**). Conversely, Cell Growth and Death, Amino Acid Metabolism, and Energy Metabolism exhibited higher abundance in adjacent nontumor tissues. While Carbohydrate Metabolism displayed greater prevalence in cancer tissues compared to adjacent group, the disparity lacked significance. This discrepancy may stem from the limited sample size and pronounced inter-group variations among clinical samples. For a more comprehensive elucidation of metabolic pathways, please refer to **Supplementary Table 4**.

### 3.6 Microbial differences in nicotine exposure and tumor progression

**Figure 5:**
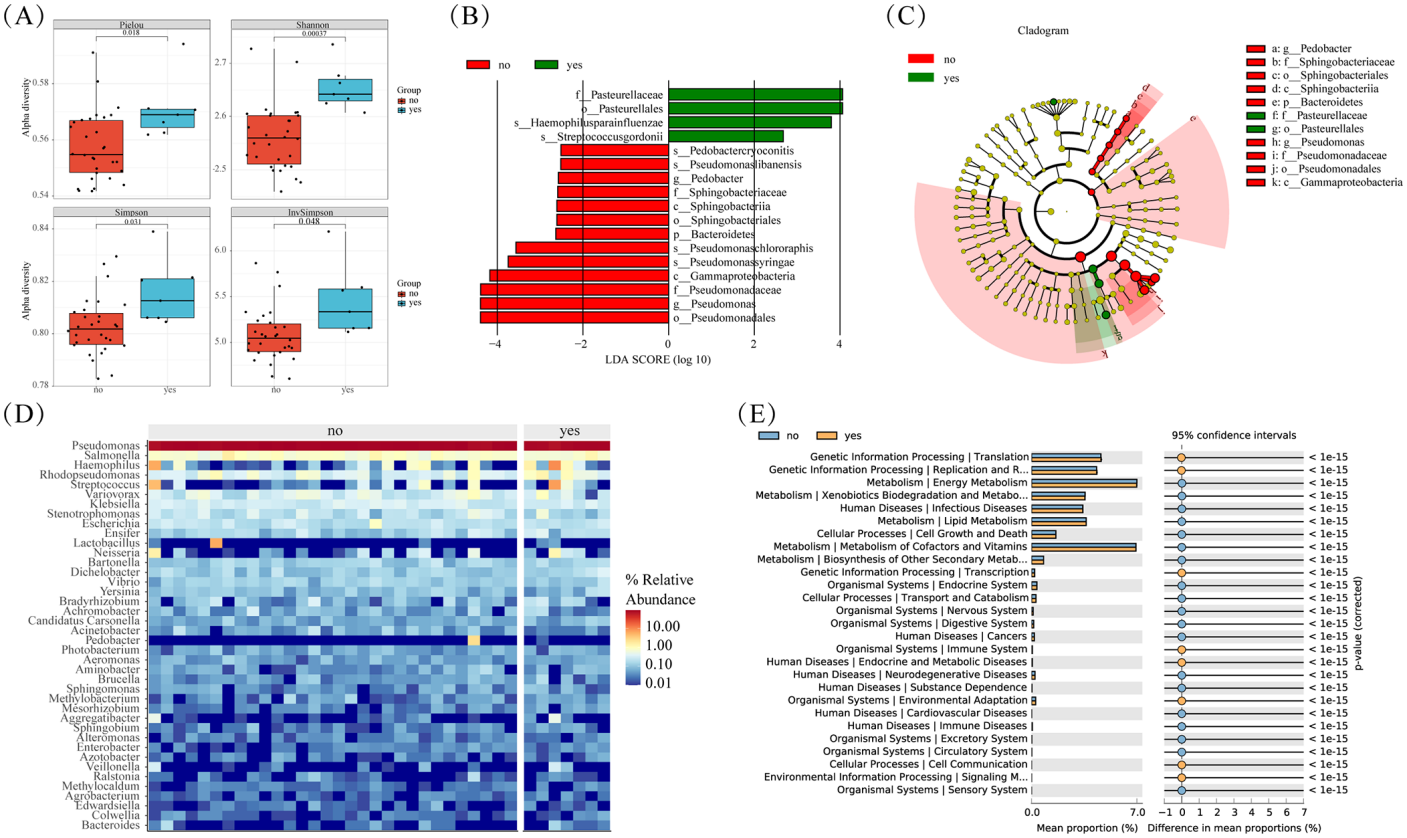
Microbial differences between smoke and non smoke groups. **(A)** Box plots of differences in alpha diversity index between smoke and non smoke groups. **(B)** Caldogram of differential bacterial taxa from the phylum to the genus level. **(C)** LDA histogram of differential microbiota at the genus level. **(D)** Heatmap of relative abundance of the top 20 microorganisms at the phylum and genus levels. **(E)** Significantly enriched metabolic pathways by p-value<=0.05 in different groups.

Smoking stands out as a primary risk factor for lung cancer. Within tobacco smoke lie numerous carcinogens capable of inducing mutations and damage cellular DNA. These alterations trigger profound shifts within normal cells and the tumor microenvironment (TME), thereby fostering cellular carcinogenesis. Our study delved into the microbial diversity variances between smoking and non-smoking cohorts. Although the P-value didn’t reach significance, we observed a notable trend towards elevated tumor microbiota abundance in the smoking cohort when compared to non-smokers (**Figure 5A**). Differential analysis revealed that compared to non-smoking groups, the significantly enriched microorganisms in the smoking and non-smoking groups were: *Pasteuralaceae*, *Pasteuralales*, *Haemophilus paraainfluenzae*, and *Streptococcus gordonii* (**Figures 5B, C**). The clustering heatmap (**Figure 5D**) illustrated a high similarity in microbial abundance between the smoking and non-smoking groups, potentially attributed to malignant transformations in lung tissue and marginal differences in the tumor immune microenvironment.

Further insights from KEGG enrichment analysis and differential microbial community analysis reveal significant enrichment of processes such as Translation, Replication, and Repair in the smoking group (**Figure 5E**). This suggests a potential molecular basis contributing to the observed disparities in microbial composition between smoking.

**Figure 6:**
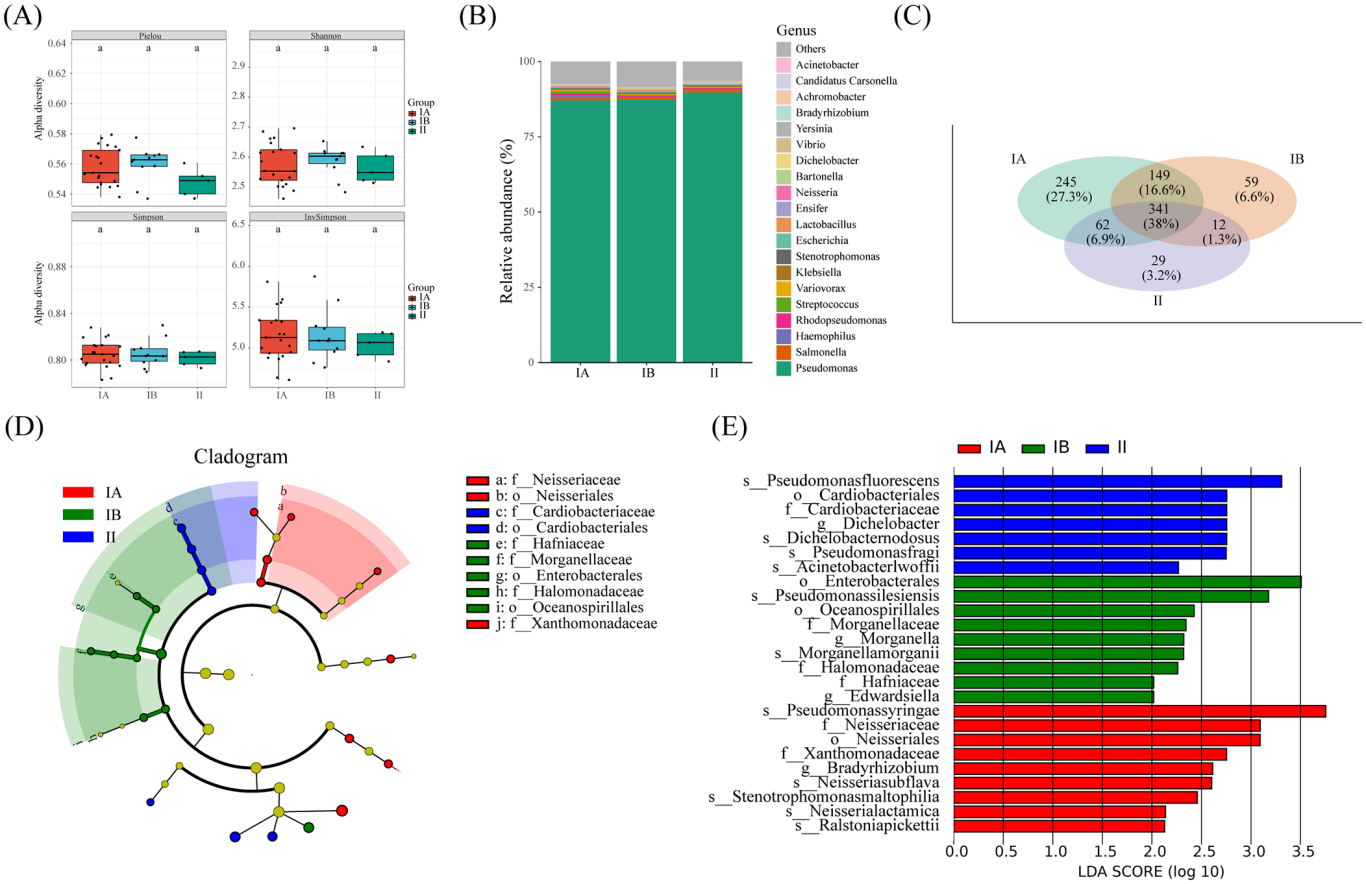
Microbial and metabolic pathways differences at different clinical stages. **(A)** Box plots of differences in alpha diversity index between different stages. **(B)** the proportion of microorganisms at genus level. **(C)** Veen diagram shows OTUs of different stages. **(D)** Caldogram of differential bacterial taxa from the phylum to the genus level. **(E)** LDA histogram of differential microbiota at the genus level.

We conducted an in-depth analysis of microbial diversity across stages of tumors. Interestingly, 341OUT were found to be shared among tumor tissues of different stages, as depicted in **Figure 6C**. Furthermore, the microbial species composition demonstrated striking similarities across the three distinct groups, as illustrated in **Figure 6B**. Notably, no significant disparity surfaced in the alpha diversity index of microorganisms within lung cancer tissues of differing stages, as depicted in **Figure 6A**.

The results from LEfSe analysis unveiled the distinctive enrichment patterns of microorganisms within tumor tissues of different stages. Specifically, there was a significant enrichment of the microbiota belonging to *Cardiobactereae* and *Cardiobacterales* in stage II, while *Nosseriaceae* and *Nosseriales* exhibited significant enrichment in stage IA (**Figures 6D, E**). This nuanced insight into the microbial landscape within varying tumor stages enriches our understanding of the intricate relationship between the microbiome and the progression of lung cancer.

## 4 Discussion

The human lung, known for its expansive surface area, plays a vital role in facilitating the exchange of oxygen and carbon dioxide. With the development and application of high-throughput sequencing technology, it has been confirmed that there are microbial communities present in both healthy and diseased lungs (Harris et al., 2007; Charlson et al., 2011). However, the cultivation of these microorganisms remains a challenge, with less than 20% currently amenable to laboratory cultivation (Venkataraman et al., 2015). Numerous studies have explored the correlation between the lung microbiota and lung cancer. However, these investigations encounter limitations stemming from variations in sampling conditions, experimental procedures, environmental pollution, and other confounding factors. Given the direct connection between the lungs and the external environment, the presence of environmental bacteria interfering with lung samples poses a significant hurdle to research in this field. Presently, conventional methods such as surgical resection and chemotherapy serve as the primary treatment approaches for lung cancer. However, the link between the symbiotic microorganisms within tumor tissue and the immune microenvironment of the tumor remains elusive. Consequently, extensive research into the mechanisms through which microorganisms contribute to lung cancer progression and the microbial communities associated with tumor development assumes paramount importance. Such research endeavors lay the foundation for harnessing microorganisms as potential targets for therapeutic intervention in the treatment of tumors.

In this study, we obtained tumor samples along with paired adjacent nontumor tissues from surgical patients initially diagnosed with lung adenocarcinoma. Employing nanopore sequencing, we conducted high-throughput sequencing on bacteria, yielding sequences with full length of 16s. Our findings revealed that the top five bacteria, dominating at the phylum level, were *Proteobacteria*, *Firmicutes*, *Bacteroidetes*, *Actinobacteria*, and *Cyanobacteria*. Remarkably, these results align with prior research (Erb-Downward et al., 2011; Gomes et al., 2019). At the genus level, *Pseudomonas*, *Haemophilus*, *Salmonella*, *Rhodopsudomonas*, and *Escherichia* exhibited higher abundance. Notably, *Salmonella* and *Rhodopsudomonas*, uncommon in pulmonary microecology studies, were detected in our experiment, suggesting potential sampling or environmental bacterial contamination.

Alpha diversity analysis indicated a significant reduction in bacterial alpha diversity within tumors compared to adjacent tissues. Studies have indicated that the epithelial cell states and plasticity in early-stage lung adenocarcinoma (LUAD) are associated with various malignant cell states and are closely linked to LUADspecific oncogenic drivers (Han et al., 2024). The reduction in α-diversity may be correlated with immunosuppressive and metabolic alterations in the tumor microenvironment, which could facilitate the progression of the tumor. The microbial composition within tumor tissues has been observed to differ significantly from that in normal lung tissues, with notable changes in the abundance of certain microbial groups. These variations may be related to the early development and progression of the tumor and could potentially influence patients’ therapeutic responses and prognosis (Yu et al., 2016; Greathouse et al., 2018). Beta diversity results demonstrated higher diversity in adjacent tissues along both PCOA1 and PCOA2 axes. The PERMANOVA analysis yielded a p-value of 0.001, signifying a notable difference in microbial composition between cancerous and adjacent tissues. Furthermore, the differential analysis via LEfSe unveiled a significant enrichment of *Streptococcaceae*, *Neisseriaceae*, and *Pasteurellaceae* in the tumor group. This observation was corroborated by non-parametric tests (rank sum tests), reaffirming the distinct microbial landscape associated with lung adenocarcinoma. *Streptococcaceae* and *Neisseriaceae* are intricately linked to respiratory ailments like chronic lung infections. Several studies suggest that heightened *Neisseriaceae* levels correlate with tumor occurrence and progression. Meanwhile, Salvador Bello’s findings indicated a significant elevation in *Streptococcaceae* abundance at center of cancer, presenting a composition notably distinct from control groups. *Streptococcaceae* emerges as a potential biomarker for lung cancer screening (Bello et al., 2021; Song et al., 2022). However, what intrigued us was the elevated abundance of *Lactobacillaceae* in the tumor group compared to adjacent tissues. This observation mirrors a study by Lauren et al. on cervical cancer, where they found that L-lactate-producing substances induce chemotherapy and radiation resistance in cervical cancer cells, prompting metabolic recombination or alterations in multiple metabolic pathways within the TME (Colbert et al., 2023).

Simultaneously, we delved into the relationship between tumor microbiota, nicotine exposure, and various stages of tumor progression. Overall, the alpha diversity of the microbiota in the nicotine-exposed group surpassed that of the non-exposed group, even though the p-value did not reach statistical significance. Differential analysis revealed that non-smoking group exhibited microbial enrichment primarily dominated by *Pseudomonas*, *Sphingobacteriaceae*, and *Gammaproteobacteria*. Research indicated that increased *Pseudomonas* abundance benefits the body by enhancing immune resistance against tumor cell invasion. Additionally, *Gammaproteobacteria* have been tentatively associated with drug resistance in pancreatic ductal adenocarcinoma. The prevalence of *Sphingobacteriaceae* in pancreatic ductal adenocarcinoma correlates with prolonged patient survival (Chang et al., 2014; Geller et al., 2017; Riquelme et al., 2019). Moreover, we examined microbiota variances across various tumor stages and observed minimal differences in alpha diversity among them. However, notable distinctions emerged in the enriched microbiota across tumor stages. *Neisseriales* showed significant enrichment in stage IA, *Enterobacteriales* predominated in stage IB, and *Cardiobacteriales* emerged as significantly enriched in stage II. Limited research exists on the association between this bacterium and tumors. Its significant enrichment in stage II suggests a potential link to the weakened immune system of patients at this stage. Moreover, the low abundance of *Cardiobacteriales* might proliferate into the primary differential microbiota.

The functional enrichment analysis of microorganisms revealed that pathways significantly enriched in the tumor group included Folding, Sorting and Degradation, Transcription, Immune Diseases, and Cell Communication. These pathways are intricately linked to tumor cell proliferation and TME. In contrast to previous studies highlighting the increased transfer of carbon to fatty acids in cancer cells for membrane and signal molecule biosynthesis, our study did not find enrichment in pathways related to lipid metabolism in the tumor group (DeBerardinis and Thompson, 2012). One plausible explanation could be the unique aerobic environment of the lungs, characterized by frequent gas exchange between cells. In this context, the energy needed for the rapid proliferation of tumor cells may be directly acquired through the tricarboxylic acid (TCA) cycle, eliminating the necessity for fatty acid oxidation to generate compensatory energy. Additionally, KEGG pathways such as Transcription, Translation, Reproduction and Repair, Endocrine and Metabolic Diseases, and Immune Diseases were significantly enriched in the nicotine-exposed group. Notably, Energy Metabolism and Lipid Metabolism were enriched in the non-nicotine.

The oxygen-rich environment of lung cells enables these microorganisms to exert their influence on the clinical characteristics of patients in distinctive ways. In general, the interaction between the microbiota linked to lung cancer and immune cells shapes the onset and advancement of lung cancer through diverse mechanisms. Owing to constraints within our experimental setup, further investigations regarding the interaction mechanisms between microorganisms and immune cells have not been pursued. Nonetheless, we contend that our findings lay a groundwork and offer direction for future exploration in this domain.

## 5 Conclusion

This study employed sequencing analysis to examine microorganisms in human lung adenocarcinoma and adjacent tissues, with the objective of pinpointing microbial communities linked to tumor onset, advancement, or nicotine exposure. Our findings revealed noteworthy distinctions in microbial compositions between lung adenocarcinoma and adjacent tissues, alongside distinctive variations in microbial communities associated with nicotine exposure and different tumor stages. The elevated presence of *Bacteroidetes*, *Neisseria*, and *Enterobacteriales* in lung adenocarcinoma suggests potential implications for the occurrence and progression of pulmonary epithelial cell carcinoma via diverse mechanisms. Due to RNA integrity issues, transcriptomic analysis of lung tissue was not conducted. Potential tumor-specific microbes were identified, but further research is needed to explore their relationships with lung tissue cells and the tumor immune microenvironment.

## Data Availability

The data presented in the study are deposited in the GEO repository, accession number GSE262090.
