# Supplementary figures and images for: Unveiling microbial dynamics in lung adenocarcinoma and adjacent nontumor tissues: insights from nicotine exposure and diverse clinical stages via nanopore sequencing technology
Source: Front Cell Infect Microbiol. 2024 Aug 27;14:1397989. doi: 10.3389/fcimb.2024.1397989 (PMC11385298; doi:10.3389/fcimb.2024.1397989)

Alpha diversity

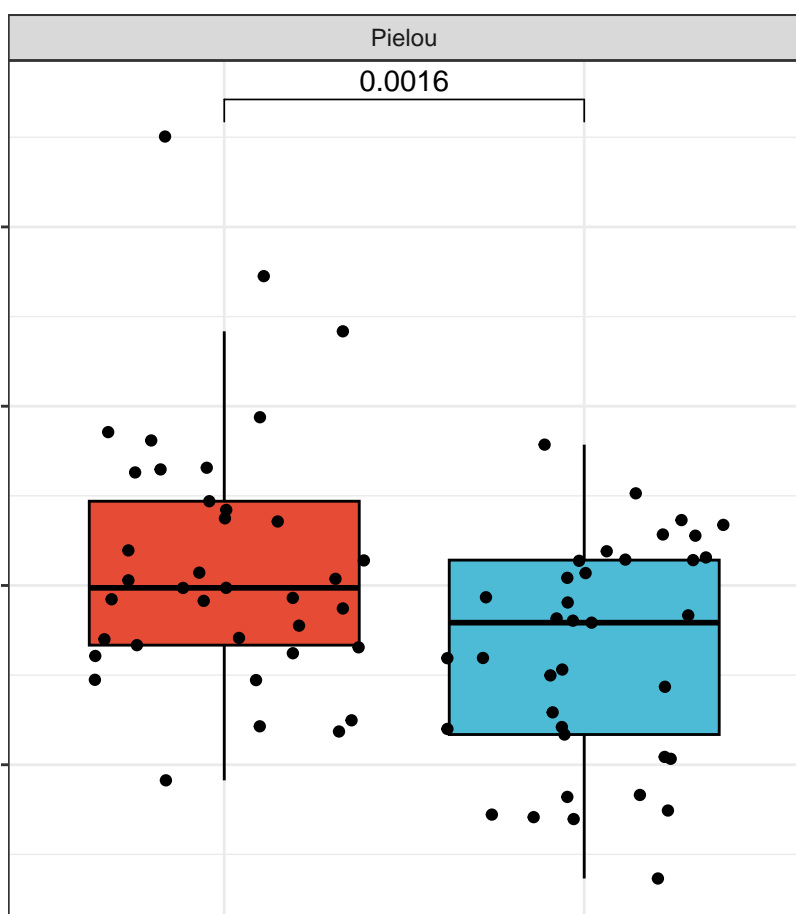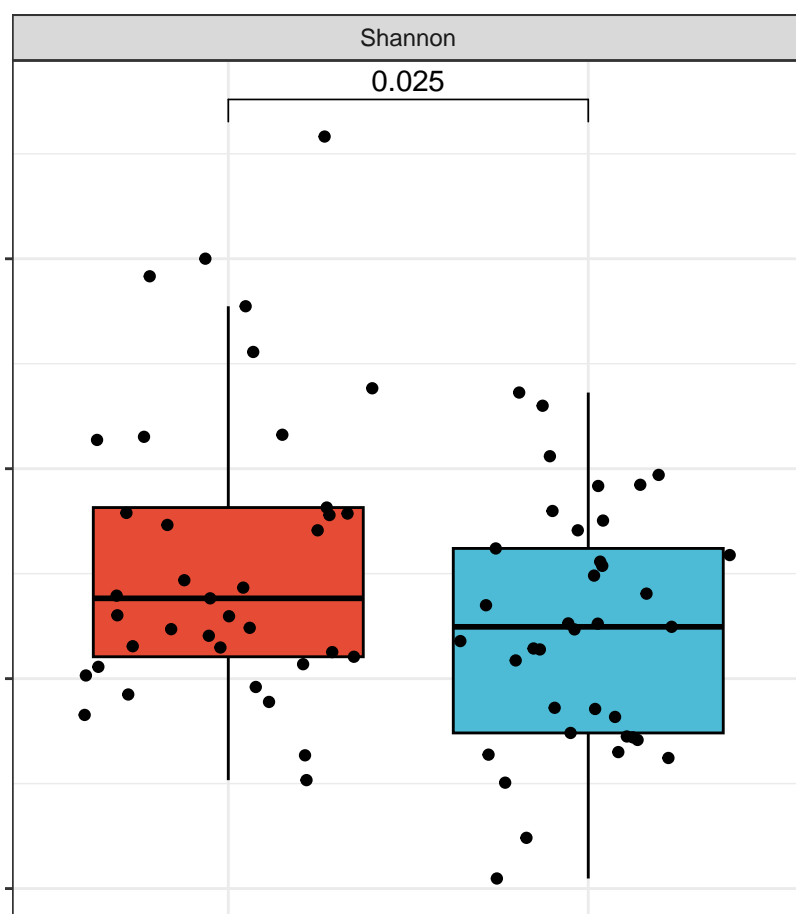

Group

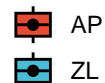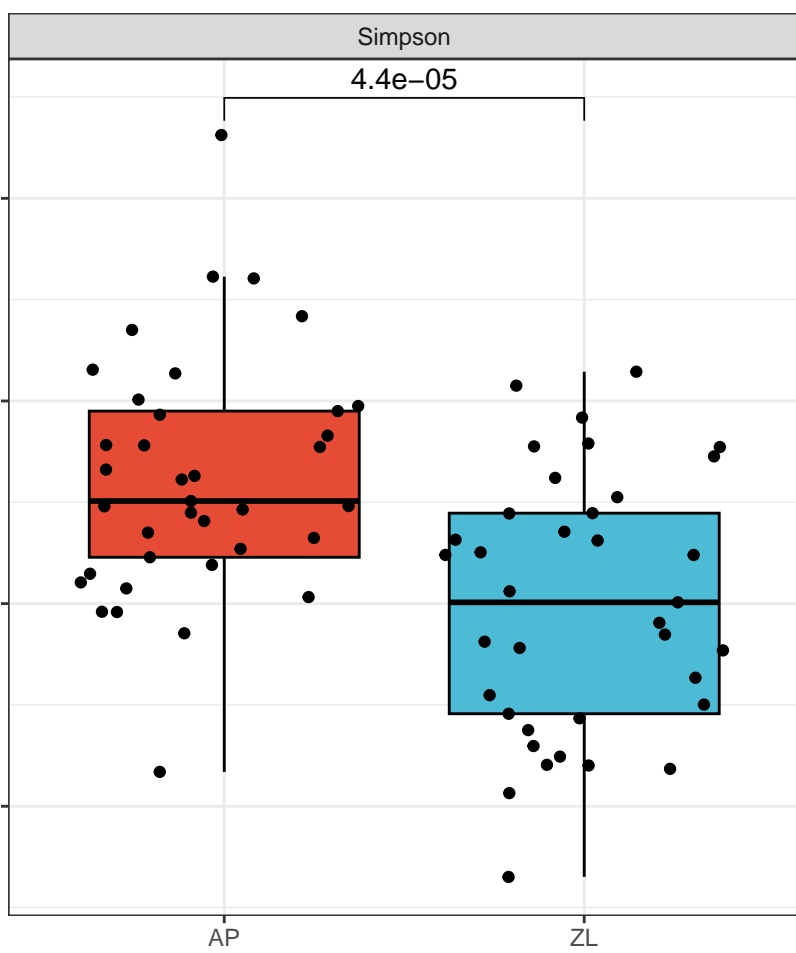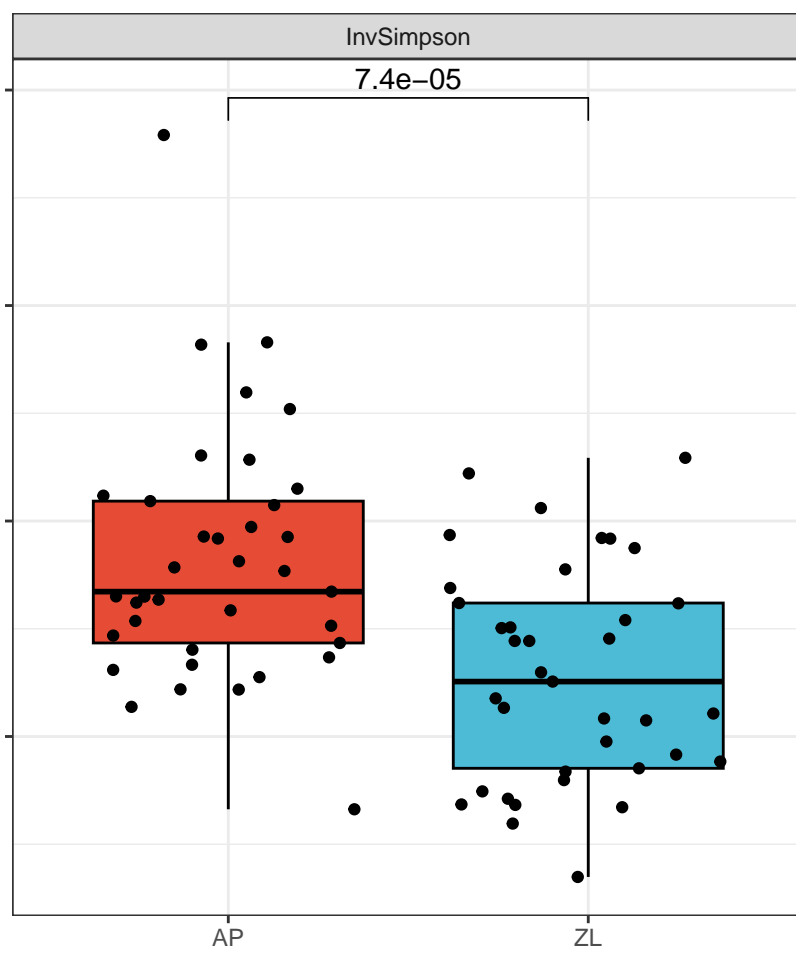

Supplement: Supplementary file 1 [file DataSheet1.pdf]
