# Supplementary material for: Unveiling microbial dynamics in lung adenocarcinoma and adjacent nontumor tissues: insights from nicotine exposure and diverse clinical stages via nanopore sequencing technology
Source: Front Cell Infect Microbiol. 2024 Aug 27;14:1397989. doi: 10.3389/fcimb.2024.1397989 (PMC11385298; doi:10.3389/fcimb.2024.1397989)
Supplement: Supplementary file 3 [file Table3.docx]

| **Characteristic** | **N = 38*^1^*** | **Characteristic** | **N = 38*^1^*** |
| --- | --- | --- | --- |
| age | 59 (54, 65) | RET | 1 (3.1%) |
| gender |  | Unknown | 6 |
| F | 25 (66%) | BRAF | 1 (3.1%) |
| M | 13 (34%) | Unknown | 6 |
| smoke | 8 (21%) | HER2 | 1 (3.1%) |
| stage |  | Unknown | 6 |
| ⅠA | 22 (58%) | CEA | 1.66 (1.36, 2.43) |
| ⅠB | 11 (29%) | Unknown | 23 |
| Ⅱ | 5 (13%) | Cyfra211 | 1.95 (1.52, 2.78) |
| location |  | Unknown | 23 |
| lower | 14 (37%) | NSE | 11.1 (10.4, 13.7) |
| upper | 24 (63%) | Unknown | 23 |
| antibiotic |  | CA724 | 2.51 (1.89, 5.55) |
| Aminoglycosides | 5 (13%) | Unknown | 25 |
| Cephalosporins | 32 (84%) | TKI | 0.97 (0.65, 1.35) |
| Penicillin | 1 (2.6%) | Unknown | 16 |
| KRAS | 3 (9.4%) | WBC | 6.7 (5.4, 11.2) |
| Unknown | 6 | PLT | 188 (167, 235) |
| EGFR | 22 (69%) | D.D | 0.24 (0.18, 0.64) |
| Unknown | 6 | Unknown | 1 |
| ROS1 | 1 (3.1%) | IL.6 | 4 (2, 6) |
| Unknown | 6 | Unknown | 26 |
| *^1^* Median (IQR); n (%) | |  |  |
