# Supplementary material for: Unveiling microbial dynamics in lung adenocarcinoma and adjacent nontumor tissues: insights from nicotine exposure and diverse clinical stages via nanopore sequencing technology
Source: Front Cell Infect Microbiol. 2024 Aug 27;14:1397989. doi: 10.3389/fcimb.2024.1397989 (PMC11385298; doi:10.3389/fcimb.2024.1397989)
Supplement: Supplementary file 6 [file Table6.docx]

Source data

My manuscript "Unveiling microbial dynamics in lung adenocarcinoma and adjacent nontumor tissues: Insights from nicotine exposure and diverse clinical stages via Nanopore Sequencing Technology" .

I have uploaded and reviewed the original data.

My Series record GSE262090 provides access to all of my data. For information on GEO linking and citing, please refer to:

https://www.ncbi.nlm.nih.gov/geo/info/linking.html.
